# GArNet: A Genetic Algorithm-Based Protein Redesign Approach to Optimize Mutation Combinations Informed by Network Theory

**DOI:** 10.1021/acs.jcim.5c00624

**Published:** 2025-06-05

**Authors:** Hiroki Ozawa, Shoryu Fujita, Taichi Chisuga, Shogo Nakano

**Affiliations:** Graduate Division of Nutritional and Environmental Sciences, 13140 University of Shizuoka, 52-1 Yada, Suruga-ku, Shizuoka 422-8526, Japan

## Abstract

Protein redesign is an enzyme engineering strategy that enhances a template enzyme’s properties by introducing mutations. Although the method is broadly employed for enzyme design, finding optimal mutation combinations with high reproducibility within a limited time frame remains a major technical hurdle. In this study, we report GArNet, a Genetic Algorithm-based protein redesign approach to optimize mutation combinations informed by Network theory. GArNet uses a template structure and its homologous sequences to generate mutants of which their combinations are optimized in two phases. In Phase I, repeated virtual evolution processes produce “complete network data”, in which nodes and edges represent introduced mutations and their co-occurrence, respectively. These data are merged into a single mutation network. In Phase II, the network is converted into a scale-free mutation network, and the high-centrality nodes (mutations) are selected to produce a list of mutation candidates and a mutated structure. We benchmarked GArNet using two distinct enzymes—*S*-hydroxynitrile lyase (*S*-HNL) and NAD^+^-dependent l-threonine 3-dehydrogenase (TDH). Computational analysis indicated that, under three independent runs, GArNet achieved >73.5% mutational reproducibility in *S*-HNL design, surpassing the 25% achieved by a conventional method. Experimental tests demonstrated that GArNet generated active *S*-HNLs and TDH mutants with enhanced thermostability and soluble expression compared to native ones. Taken together, these findings indicate that representing introduced mutations and their co-occurrence as a network effectively identifies beneficial mutations. GArNet is available at https://github.com/shognakano/GArNet.

## Graphical abstract

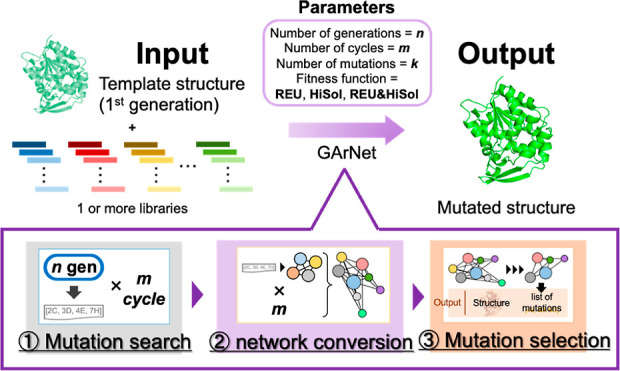

## Introduction

All species acquire organic compounds from their environment and transform them into various bioactive molecules through metabolic processes, where enzymes serve as key catalysts.^1^ Enzymes operate with high efficiency under water conditions at normal temperature. Some enzymes also exhibit strict substrate and reaction specificity (e.g., enantioselectivity and control of molecular-weight distribution of polymers), making them invaluable for synthesizing enantiomerically pure chiral compounds^2–4^ and biopolymers (e.g., biodegradable polymers^5,6^ and RNA-based therapeutics^7^). Due to these properties, enzymes have the potential to serve as next-generation biocatalysts that complement conventional chemical catalysts.^8^ However, the low stability, limited producibility, and fragility of many native enzymes have hampered significant challenges to apply them in the synthesis.^9^

To address these limitations, many research groups are now developing computational strategies to engineer the enzymatic properties. Two major strategies have been available: de novo protein design and protein redesign.^10,11^ Their key distinction is whether structures or amino acid sequences are utilized as templates in the design. In de novo design, entirely new proteins with specified structures and functions are generated without referencing the template as input data.^11^ Several research groups have leveraged deep learning-based tools (e.g., ProteinMPNN^12^ and RFDiffusion^13^) to generate de novo proteins with enzymatic activity. For example, ProteinMPNN, which solves the inverse folding problem, is particularly effective for enzyme engineering to enhance thermostability and producibility as it can generate multiple protein sequences from the specified structural data.^14,15^ On the other hand, protein redesign is a more traditional strategy that aims to improve template enzyme’s properties by introducing beneficial mutations.^11^ The protein redesign approach tends to increase desirable properties with minimal trade-off in intrinsic activity.^7^ In this study, we focus on advancing protein redesign.

Protein redesign can be classified into the following two groups: sequence-based or structure-based design. In sequence-based design, mutants are generated via statistical analyses of homologous sequences—commonly through consensus design^16,17^ or ancestral sequence reconstruction.^18–20^ These techniques require only a sequence library, making them computationally cost-effective with high generality. Success often depends heavily on user expertise, particularly in curating the homologous sequences, which should be performed prior to the design becomes knowledge-intensive.^21^ In contrast, structure-based approaches rely on the structural data to produce mutants with enhanced stability, which can be estimated by computational tools. Tools such as Rosetta^22^ and FoldX^23^ use protein structures to deliver more reliable designs, albeit with higher computational costs.

Hybrid methods that combine sequence- and structure-based designs have emerged, with PROSS^24^ and FireProt^25^ now widely used to engineer highly functional enzymes. The method can narrow the sequence space for identifying highly functional enzymes by drawing on phylogenetic information encoded in the sequences, and it introduces mutations into the template with high accuracy by referencing structural data. Our group has also developed GAOptimizer,^26^ which proceeds in two steps: (1) mutations identified by multiple sequence alignment (MSA) of the template and its homologous sequence library are introduced into the template structure with PyRosetta,^27^ and (2) genetic algorithms (GA) optimize the resulting combinations using Rosetta energy units (REU) as a fitness function. Although the GAOptimizer can generate multipoint mutants with optimized mutation sets, low reproducibility of the mutants would be a technical hurdle for the application; this likely arise from the metaheuristics of GA.^28^ Increasing the number of generations (>300) could improve reproducibility but becomes computationally expensive.^26^ Moreover, a previous study showed that mutants selected before REU scores reached full convergence displayed better enzymatic properties than those chosen after full convergence.^26^ Hence, developing a new algorithm that reliably produces advantageous mutants in previous generations is essential. Herein, we developed GArNet, an algorithm designed for high reproducibility by combining bagging analysis of the GAOptimizer with network theory to unify mutation data. We benchmarked GArNet computationally and experimentally on two distinct enzymes—a cofactor-independent *S*-hydroxynitrile lyase (*S*-HNL) and an NAD^+^-dependent l-threonine 3-dehydrogenase.

## Results

### Algorithm of GArNet

GArNet, a Genetic Algorithm-based protein redesign approach informed by Network theory, requires two inputs: (1) a PDB file of the target protein (template structure in Figure 1) and (2) more than one library of homologous sequences in the FASTA format; the library includes from several to hundreds of sequences (Figure 1). Before running GArNet, the template structure should be energy-minimized. The homologous sequences can be identified through BLASTp.^29^ In addition to these inputs, users must specify four parameters: the number of cycles (*m*), the number of generations (*n*), the number of mutations (*k*), and the fitness function which would determine what types of mutations are selected (REU, HiSol score, or both REU & HiSol score [REU + HiSol]) (Figure 1). REU estimates the stability of a given 3D protein structure, whereas the HiSol score evaluates the hydrophobicity discrepancy between the template sequence and the library, as defined in a previous study.^26,30^ GArNet introduces those mutations into the template that are phylogenetically confirmed within the homologous sequences, as identified through an MSA of the template and library. Using these inputs and parameters, GArNet generates a list of mutation candidates and the mutated structure through two phases of calculation (Figure 1). In Phase I, the mutation network was constructed as following procedures. First, total *n* generations of virtual evolution were performed by the GAOptimizer.^26^ The elite structure at the *n*th generation (the “lastgen-elite” in Figure 1) is compared with the template, and any introduced mutations are saved as a “complete network” data (Figure 1). Then, this process is repeated for *m* cycles. When REU or HiSol score was used as the fitness function, mutations are accumulated into the template protein during the total *m* of the evolutionary process, following the procedure described previously. In contrast, when both REU and HiSol (REU + HiSol) were adopted, GArNet executes half of the *m* cycles under REU and the remaining cycles under HiSol. Finally, the total *m* of complete network data sets are merged into a single mutation network (single network in Figure 1) by summation, where nodes represent confirmed mutations (e.g., the substitution of residue 4 to Glu is labeled as “4E”) and edges represent co-occurrence of these mutations during virtual evolution. Node and edge data are saved in Node count and Edge count files separately (Figure 1). In the node count file, “1A”:5 indicates that the 1A mutation appeared five times over the total *m* cycles of the GAOptimizer run (Figure 1). In the edge count file, (“1A”,“2C”):1 means that the 1A–2C co-occurrence was detected once during these *m* cycles (Figure 1). The number of *n* and *m* affects the reproducibility of the selection of mutation candidates; this point will be discussed in the latter section.

**Figure 1:**
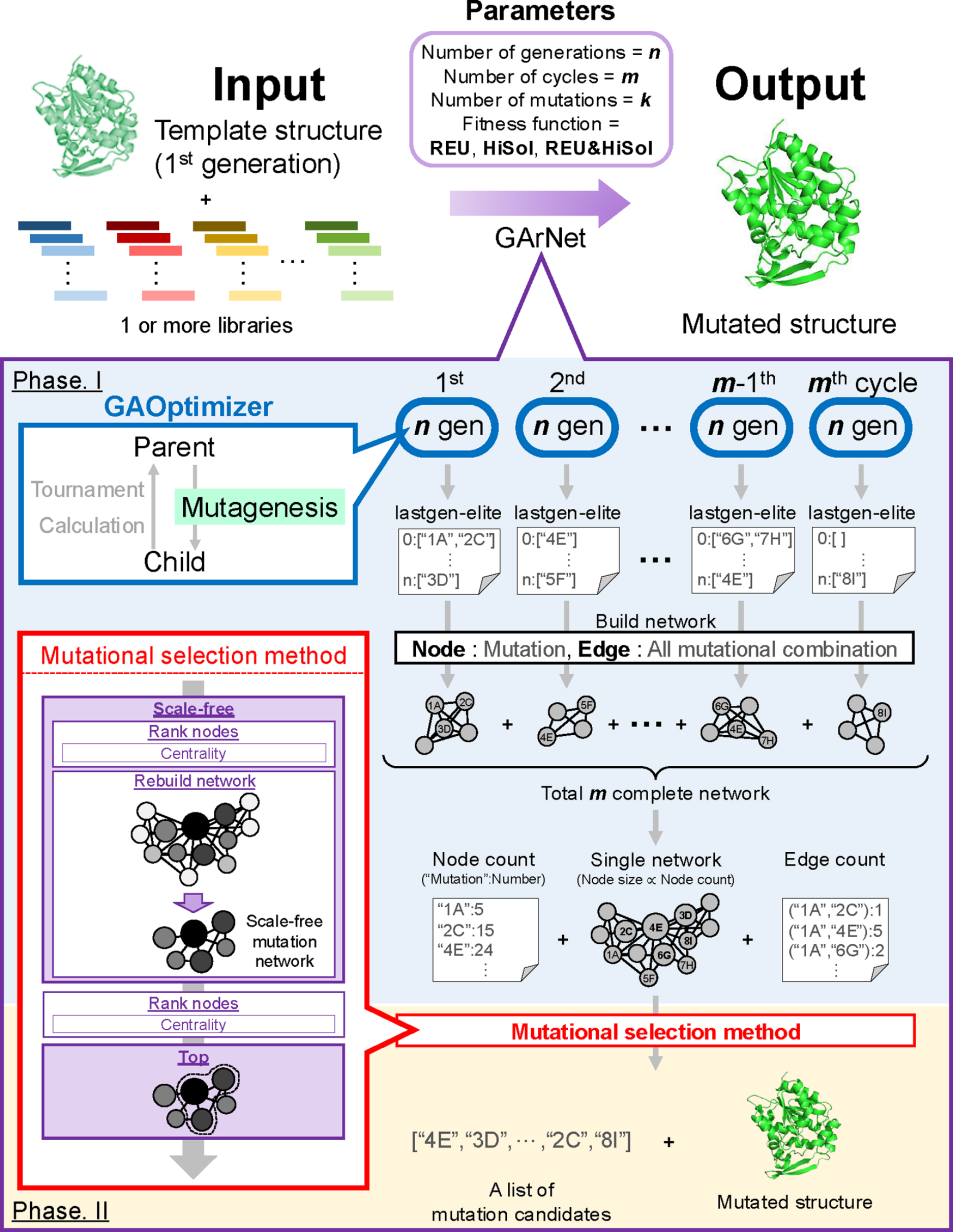
Schematic view for algorithms of GArNet. GArNet produces two output files: a list of mutation candidates and a mutated structure. Multiple cycles (total *m*) of the GAOptimizer are performed, and the introduced mutations in each cycle are recorded as a complete network in which nodes represent mutations and edges indicate their co-occurrence. Total *m* of complete networks are merged into a single mutation network, from which the list of the mutations and mutated structure are generated by the mutation selection method.

In Phase II, the list of mutation candidates and the mutated structure (Figure 1) were generated via the mutational selection method (Figure 1). GArNet converts the single network into a scale-free mutation network via the rebuild network process (Figure 1) using either a descending or an ascending approach for curation of the network (Figure S1). In the descending approach, nodes with high centrality (high-rank nodes in Figure S1) are selected, and edges are drawn between the selected nodes with referring to the edge count file (Figure 1); this process is repeated until the network’s exponent of power law (*g*-value) converges to 2–3 (Figure S1). In the ascending approach, nodes with low centrality (low-rank nodes in Figure S1) are removed, along with any associated edges (Figure S1), and this pruning continues until the *g*-value reaches 2–3 (Figure S1). Here, other researchers have hypothesized that, in scale-free networks, the *g*-value typically lies between 2 and 3.^31^ Through several trials, we adopted the ascending approach to minimize information loss compared with the descending approach; in fact, for the case of design of GanHNL-Hi5p, the remaining mutations after the curation by adopting an ascending approach (228 in Figure S1) were >2-fold larger than that of a descending approach (99 in Figure S1). From this scale-free mutation network, the program then identifies the top *k* nodes bearing high centrality (mutations), saving them as a list of mutation candidates (Figure 1). These mutations are introduced into the template structure, and both REU and HiSol score^26,30^ are calculated (Figure 1). By specifying an additional parameter, the program can select *k* ± *i* (where *i* is an integer) mutations and propose the top candidates showing the greatest improvement for the score. Prior to ordering synthetic genes that encode the GArNet-designed mutants, comparison of the mutated structure with the template structure (Figure 1) is recommended to identify—and thus avoid introducing—false-positive mutations that affect negatively enzymatic properties. The more detailed information, including classes and functions of GArNet used in these steps, is described in the Supporting Information.

### Benchmark Analysis of GArNet through Design of *S*-Selective Hydroxynitrile Lyases

In the previous section, we introduced the GArNet algorithm. Here, we evaluated its utility in enzyme engineering by enhancing the properties of the *S*-hydroxynitrile lyase (*S*-HNL) from *Manihot esculenta* (MeHNL).^32^ This α/β-hydrolase fold enzyme converts *S*-cyanohydrins to the corresponding aldehydes (and vice versa).^33,34^ Figure 2A illustrates, as an example, the conversion of *S*-mandelonitrile to benzaldehyde, which we used to measure the *S*-HNL activity. *S*-HNLs are widely employed to synthesize *S*-cyanohydrins—precursors for pesticides and pharmaceuticals—motivating numerous research efforts to improve their characteristics.^35,36^ The GAOptimizer was previously benchmarked on MeHNL, allowing direct comparison with GArNet and PROSS.^26^ In the previous study, GaHNL-12gen (12gen) demonstrated higher soluble expression and thermostability than MeHNL without sacrificing activity, whereas PROSS generated a mutant (PrHNL) with increased solubility at the expense of enzyme activity;^26^ here, the 12gen was selected at the 12th generation out of 500 before complete score convergence and utilizing HiSol score as fitness function. We used 12gen as a control to assess GArNet (Figure 2B). Before experimental testing, we compared GArNet with GAOptimizer computationally. The mutation reproducibility of the 12gen was only 25%, with T117V and I139N being the only shared substitutions across three trials (Table S1). In contrast, GArNet yielded higher reproducibility (Table S2). Under moderate-to-high generation and cycle settings (*n* = 10, *m* = 100), reproducibility improved to 90.0% (GanHNL-Hi10, termed Hi10) and 80.0% (GanHNL-REHi10, referred to as REHi10).

**Figure 2:**
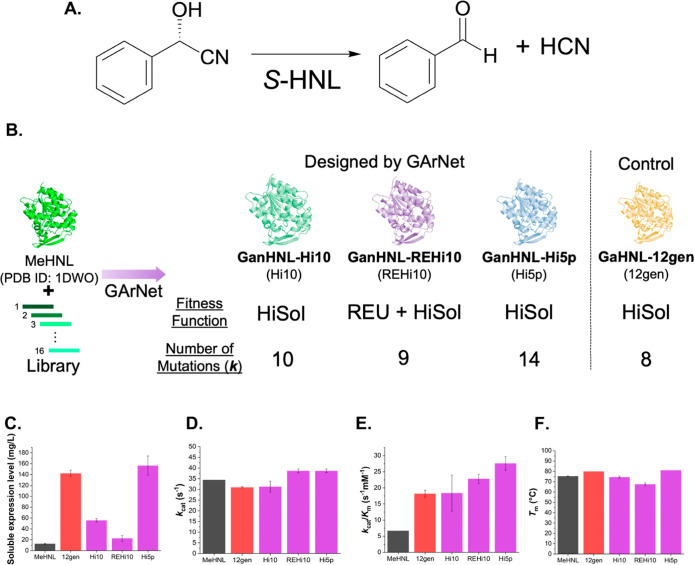
Reaction scheme of *S*-HNL (A). In this study, the activity of the designed *S*-HNLs was evaluated by measuring the time-dependent production of benzaldehyde. Schematic representation of the GArNet design process for three *S*-HNLs (GanHNL-Hi10, GanHNL-REHi10, and GanHNL-Hi5p) (B). The figure includes details of the fitness function and the number of introduced mutations. GaHNL-12gen, previously generated by GAOptimizer, was used as a control. Enzymatic properties of the *S*-HNLs (C–F). Soluble expression levels in *E. coli* (C), *k*_cat_ (D), *k*_cat_/*K*_m_ (E), and *T*_m_ values (F) are shown in the figures. Bar graphs for MeHNL (native *S*-HNL), GaHNL-12gen, and the newly designed *S*-HNLs by GArNet are shown in black, red, and purple, respectively. All of the measurements were performed independently three times (*N* = 3). The data are shown as the mean ± SD.

We then experimentally examined three *S*-HNL mutants designed by GArNet: Hi10, REHi10, and GanHNL-Hi5p (Hi5p). Table S3 provides details of the design of GanHNLs. Specifically, Hi10 and Hi5p were created using HiSol as the fitness function, whereas REHi10 was generated using REU + HiSol. Table S3 outlines the details for designing GanHNLs. Hi10 and Hi5p were created with HiSol as the fitness function, whereas REHi10 was generated by using REU + HiSol. Figure 2B outlines their design parameters, including the fitness functions and mutation counts, which finally introduced 9–14 substitutions into MeHNL (Table S3). The mutation reproducibility ranged from 73.5% to 90.0%, surpassing the 25% observed for 12gen (Table S1). Changes in REU and HiSol scores (ΔREU and ΔHiSol) showed that Hi10 and Hi5p had notably lower HiSol scores (−22.3 and −26.9, respectively) and a weak decrease of both REU scores (−11.8 and −8.0, respectively), while REHi10 achieved simultaneous decreases in both REU (−17.0) and HiSol (−18.0) (Table S3). Table S3 shows that GArNet can identify mutations not selected in 12gen. Of the 14 mutations in Hi5p, 7 which are not underlined in Table S3 were selected as beneficial by GArNet but not by the GAOptimizer. The protein sequences of GanHNLs and their identities with MeHNL are listed in Tables S4 and S5.

Next, we evaluated soluble expression levels, *k*_cat_, *k*_cat_/*K*_m_, and melting temperature (*T*_m_) for MeHNL and each mutants (Figure 2C–F and Tables S6–S8). GArNet (purple bars) produced mutants with improved properties comparable to those from GAOptimizer (red bars). Among them, Hi5p achieved the best overall profile: its soluble expression reached 156.5 mg/L, exceeding 12gen (142.3 mg/L) and MeHNL (12.8 mg/L). Although *k*_cat_ values were similar, Hi5p′s *k*_cat_/*K*_m_ (27.6 s^–1^·mM^–1^) was over 1.5- and 4.0-fold higher than those of 12gen (18.2 s^–1^·mM^–1^) and MeHNL (6.7 s^–1^·mM^–1^) (Figure 2D, E and Table S7). Hi5p also had the highest *T*_m_ (81.1 °C), slightly exceeding those of 12gen (80.0 °C) and MeHNL (75.4 °C) (Figure 2F and Table S8). By contrast, REHi10, which showed improved REU and HiSol scores, exhibited a *T*_m_ of 67.5 °C—over 7 °C lower than MeHNL (Figure 2F and Table S8)—indicating that a reduction in REU does not invariably enhance thermostability, consistent with prior findings.^37^ Computational and experimental analyses of the designed *S*-HNLs indicate that GArNet can yield highly functional enzymes with a high mutation reproducibility.

### Design of Highly Functional l-threonine 3-Dehydrogenase by GArNet

The next mission is to demonstrate the broader applicability of GArNet in enzyme engineering. Accordingly, we applied it to improve the properties of another target enzyme: l-threonine 3-dehydrogenase (TDH).^38^ TDH is an NAD^+^-dependent enzyme that catalyzes the dehydrogenation of β-hydroxyl group of l-threonine (Figure 3A).^38^ These enzymes are expressed across bacteria^39–41^ to mammals,^42^ playing central roles in threonine catabolism.^43,44^ Their high specificity for l-threonine makes them suitable for quantifying this amino acid in biological samples.^45^ Here, TDH serves as a second model to test GArNet’s generality: cofactor-dependent enzymes require more stringent mutation design than *S*-HNLs because substitutions that disrupt substrate, product, or cofactor binding will drastically reduce activity. Demonstrating improved TDHs would thus support GArNet’s potential for cofactor-dependent enzyme engineering. Additionally, our group regularly employs TDHs as model enzymes to validate new protein-engineering tools,^26,46,47^ allowing precise comparison of their properties.

**Figure 3:**
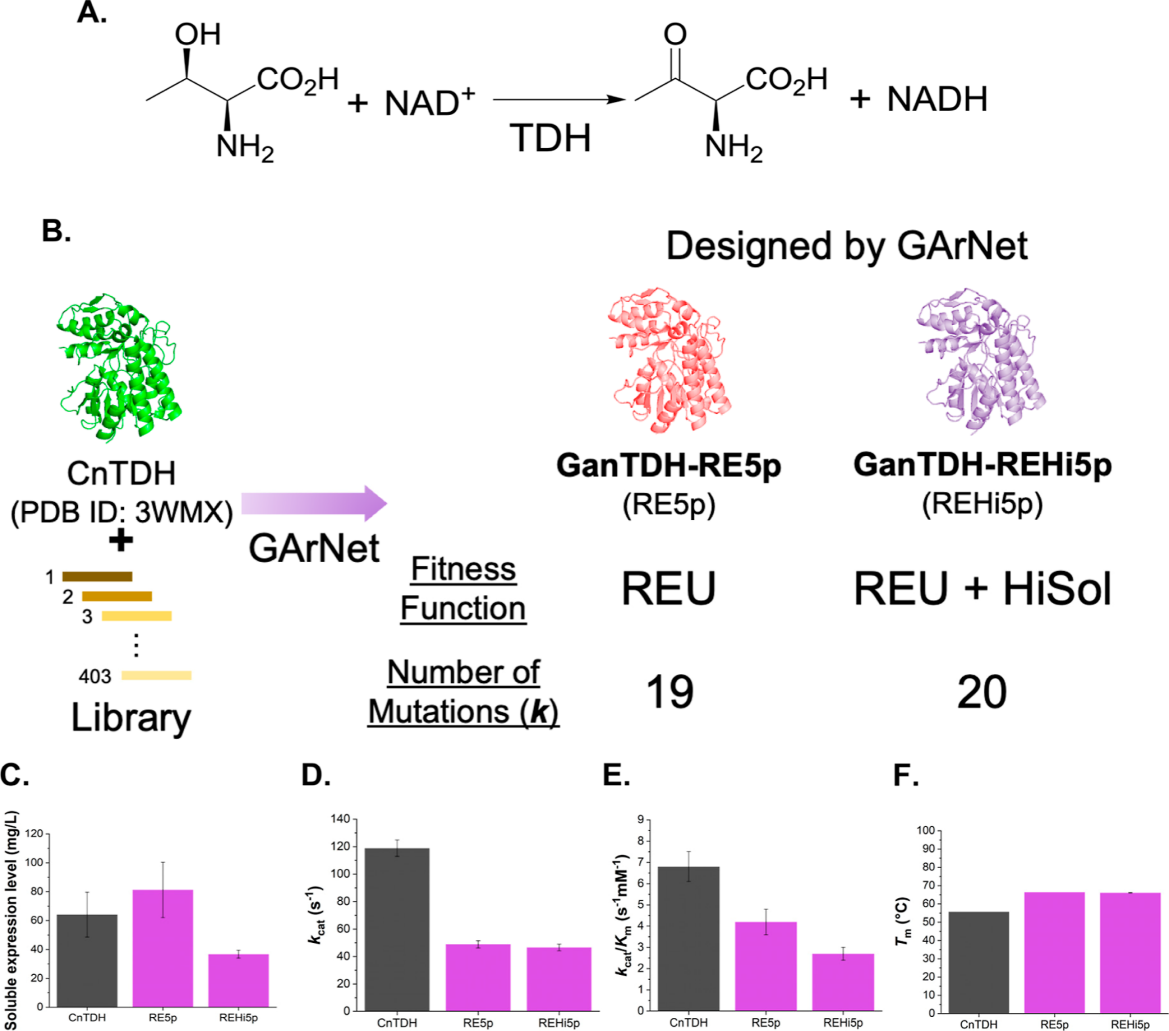
Reaction scheme of NAD^+^ dependent l-threonine 3-dehydrogenase (TDH) (A). Schematic view of how to design two TDHs (GanTDH-RE5p and GanTDH-REHi5p) by GArNet (B). The figure represents details of the design, including utilized fitness function and the number of introduced mutations. Enzymatic properties of the designed TDHs (C–F). Soluble expression level in *E. coli* (C), *k*_cat_ (D), *k*_cat_/*K*_m_ (E), and *T*_m_ values (F) are shown in the figures. Bar graphs for CnTDH (native TDH) and the designed TDHs by GArNet are shown in black and purple, respectively. All of the measurements were performed independently three times (*N* = 3). The data are shown as the mean ± SD.

Figure 3B outlines GArNet’s TDH-design procedure, using the crystal structure of TDH from *Cupriavidus necator* (CnTDH) and a library of 403 homologues (the VLYM data set) as inputs; the data set was prepared in the previous study.^48^ In the sequences of this data set, the 143rd, 174th, 188th, and 214th residues are consistent with Val, Leu, Tyr, and Met, respectively.^48^ Using these inputs, we created two GanTDHs: GanTDH-RE5p (RE5p) and GanTDH-REHi5p (REHi5p), optimized by REU or REU + HiSol fitness functions, respectively (Figure 3B). The number of introduced mutations was 19 (RE5p; Figure 3B) and 20 (REHi5p; Figure 3B), respectively. Both mutants showed reduced fitness scores (ΔREU scores of −47.4 and −43.5, and ΔHiSol scores of −27.5 and −42.1 for RE5p and REHi5p, respectively; Table S9) and high mutation reproducibility (60.0%; Table S9). The sequences of these GanTDHs and their identities relative to those of CnTDH are provided in Tables S10 and S11.

We then characterized the enzymatic properties of CnTDH, RE5p, and REHi5p, measuring soluble expression levels in *Escherichia coli* (Figure 3C and Table S12), *k*_cat_ (Figure 3D and Table S13), *k*_cat_/*K*_m_ (Figure 3E and Table S13), and *T*_m_ (Figure 3F and Table S14). A clear stability–activity trade-off emerged. Both RE5p (66.4 °C) and REHi5p (66.1 °C) displayed *T*_m_ values exceeding that of CnTDH (55.7 °C) by more than 10 °C (Table S14). However, their *k*_cat_ and *k*_cat_/*K*_m_ values were reduced to less than 50% and 70% CnTDH (118.9 s^–1^ for *k*_cat_, 6.8 s^–1^·mM^–1^ for *k*_cat_/*K*_m_; Table S13). Soluble expression levels also differed: RE5p (81.2 mg/L; Table S12) exceeded CnTDH (64.1 mg/L; Table S12), whereas REHi5p (36.8 mg/L; Table S12) was lower. Overall, these findings suggest that GArNet can successfully design more thermostable and soluble TDHs, indicating that the cofactor dependence is not an inherent barrier to this approach.

## Discussion

Successful designs of GanHNLs and GanTDHs, which surpass their native counterparts, confirm GArNet’s ability to enhance enzymatic properties. GArNet can improve the mutation reproducibility and provide more robust results than that of GAOptimizer’s design. In fact, the mutation reproducibility of an elite structure before full score convergence was quiet low (about 25%); the elite was designed by the GAOptimizer. The 12gen exemplifies this scenario and suggests that acquiring the desired mutant relies heavily on chance. Such a strategy can sample a broad mutation space—similar to deep learning-based approaches like ProteinMPNN^12^—but likely requires extensive laboratory screening for highly functional mutants. Sequence analyses reveal that the mutations accumulated in each template protein vary, depending on the chosen fitness function. Figure 4A,B shows the differences in amino acid frequencies between MeHNL and Hi5p (under the HiSol fitness function; Figure 4A), and between CnTDH and RE5p (under the REU fitness function; Figure 4B). When HiSol is used, the total number of charged residues changes minimally as the increase in Lys is offset by a corresponding decrease in Arg (Figure 4A). In contrast, applying REU as the fitness function leads to a higher number of charged residues including Lys, Asp, and Glu (Figure 4B). These substitutions occur primarily on the protein surface and may enhance thermostability by reducing aggregation.^49^

**Figure 4:**
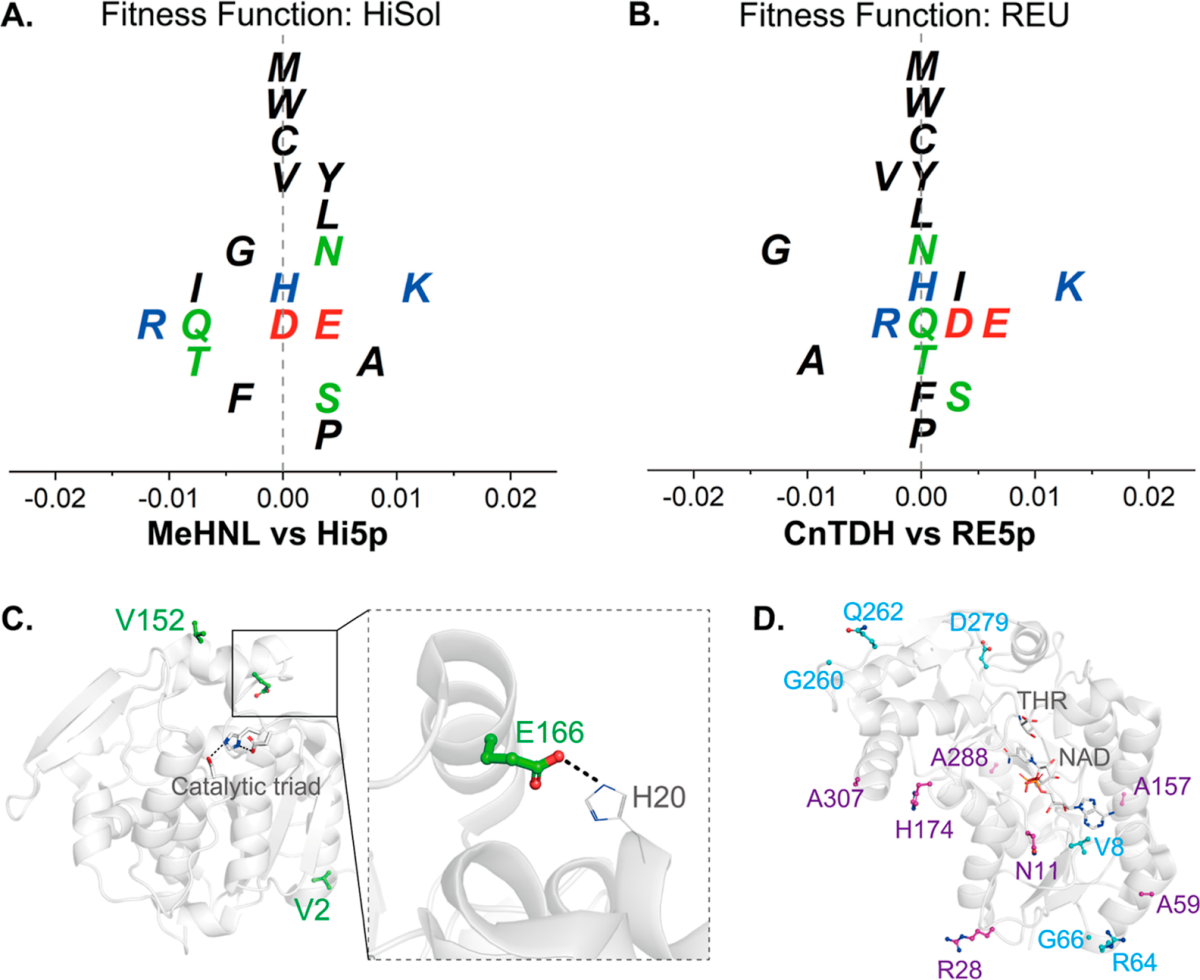
Differences in amino acid frequencies between MeHNL and Hi5p (A), and between CnTDH and RE5p (B). On the *x*-axis, positive and negative values indicate increases or decreases, respectively, in each amino acid caused by the design Mutation sites unique to Hi10 or REHi10, mapped onto the MeHNL crystal structure (PDB ID: 1DWO) (C). Mutation sites unique to RE5p or REHi5p, mapped onto the CnTDH crystal structure (PDB ID: 3WMX) (D). Mutation sites present only in RE5p and REHi5p are shown in cyan and magenta, respectively.

Analyses of the designed enzymes’ properties reveal clear functional differences (Figures 2C–F and 3C–F). By comparing their sequences and structures, we can infer why these differences arise. In the GanHNLs, Hi10 and REHi10 differ by only three residues yet display markedly different *T*_m_ values—Hi10s *T*_m_ exceeds REHi10 by ∼7 °C (Table S8). This discrepancy likely stems from V2A and V152 K in Hi10 and E166D in REHi10, with additional mutations common to both (Table S3). Two of these residues (V2 and V152; Figure 4B) lie on the protein surface, far from the catalytic triad, implying a minimal impact on stability. By contrast, E166 forms a hydrogen bond interaction with H20 (Figure 4B), so substituting it with Asp may cleave the interaction and destabilize REHi10. E166D may be a false positive for enhancing the stability. Careful checking of the template structure, such as confirmation of rotamer conformation and protonation state, prior to running GArNet could have prevented this mutation from being selected.

Among the designed TDHs, RE5p and REHi5p exhibit comparable *T*_m_ values (Figure 3C,F) but differ significantly in soluble expression—RE5p′s yield is ∼2.5 times higher than that of REHi5p (Table S9). The results suggest that enhancing the soluble expression of TDHs is governed by mechanisms distinct from those that improve *T*_m_. This difference arises from 13 distinct substitutions and may affect to expression level of TDHs: V8I, R64K, G66K, G260D, Q262E, and D279Q in RE5p and N11C, R28N, A59E, A157N, H174Y, A288K, and A307K in REHi5p (Table S9). Other changes (underlined in Table S9) are shared by both mutants. Figure 4D maps the unique substitutions (cyan for RE5p, magenta for REHi5p) across the TDH surface, indicating that four alanine residues—A59, A157, A288, and A307—are replaced by polar amino acids in REHi5p (magenta, Figure 4D) but remain unchanged in RE5p (cyan, Figure 4D). Alanine possesses the highest α-helical propensity among the 19 canonical residues (except for Pro);^50^ substituting the Ala residues may negatively affect to the folding at local helices and thereby reduce the soluble expression level of REHi5p.

## Conclusion

By designing GanHNLs and GanTDHs, we have shown that GArNet can enhance enzyme properties, such as soluble expression, thermostability, and activity. From a computational perspective, GArNet improves the mutation reproducibility by using a bagging-like approach in the GAOptimizer and employing network theory to unify mutations and their co-occurrence. Recent advances in deep learning-based protein design allow the generation of extensive sequence data sets; by using these data sets as input for GArNet, it becomes possible to more freely redesign the properties of target enzymes. GArNet can produce multimutant enzymes with superior properties; analyzing single, double, and higher-order variants generated by the tool may contribute to clarify the epistatic interactions among individual mutations which remains a long-standing research theme in enzymology.^51^ Nevertheless, several challenges remain for the broader adoption of GArNet in enzyme engineering. First, additional fitness functions are needed to generate functionally diverse enzymes. Currently, only three are available—REU, HiSol, and REU + HiSol. Analysis of residue frequencies in the designed versus template sequences revealed that, akin to consensus design,^17^ the REU-based approach tends to increase net charge (Figure 4B), a pattern not observed with the HiSol score (Figure 4A). In principle, any metric that represents a feature of the target protein can be used as a fitness function, including antigenic propensities and scores generated by deep-learning-based protein design algorithms. Incorporating new metrics would extend GArNet’s applicability to the design of highly functional enzymes. Second, the algorithm must be modified to optimize more than two scores concurrently. Although REHi10 and REHi5p displayed improvements in both REU and HiSol scores, their enzymatic performance was inferior to those of mutants designed utilizing a single fitness function (Hi5p and RE5p) (Figures 2C–F and 3C–F), suggesting that the REU + HiSol selection strategy might introduce detrimental mutations into the template. Structural comparison of the redesigned TDHs indicates that mutations selected using the REU + HiSol strategy may impair proper folding. Implementation of new function to GArNet that restricts mutation sites to specific secondary-structure elements—α-helices, β-sheets, or loops—may be helpful to overcome this hurdle. Defining a function to integrate multiple scoring metrics could also resolve this limitation by providing a more robust fitness criterion.

## Experimental Procedures

### Design of GanHNLs

Three artificial *S*-hydroxynitrile lyases (HNLs) were designed using GArNet in the following manner. The crystal structure of HNL from the *M. esculenta* (MeHNL; PDB ID: 1DWO) was downloaded, energy-minimized with PyRosetta,^27^ and used as the input data for the design. By following a previous study,^26^ 16 homologous MeHNL sequences were used as a library to select mutation candidates (Figure 2B). Utilizing these two data sets as input of GArNet, three artificial HNLs—GanHNL-Hi10 (Hi10), GanHNL-REHi10 (REHi10), and GanHNL-Hi5p (Hi5p)—were generated by lowering the HiSol score (Hi10, Hi5p) or both the REU and HiSol scores (REHi10). Detailed calculation conditions, including the input parameters (*n*, *m*, and *k*), are summarized in Table S3. Protein sequence data and sequence identity among the HNLs are shown in Tables S4 and S5.

### Overexpression and Purification of GanHNLs

Genes encoding the three artificial HNLs (GanHNL-Hi10, GanHNL-REHi10, and GanHNL-Hi5p) were synthesized by GenScript, codon-optimized for the *E. coli* expression system, and subcloned into the pET28b vector at the *Nde*I and *Xho*I sites. A stop codon was introduced at the 3′ end of each hnl genes. Plasmids encoding MeHNL were obtained from a previous study.^52^ Cultures were grown in 1 L of LB medium containing 40 μg/mL kanamycin at 37 °C until the OD600 value reached 0.6–0.8. At that point, the flasks were placed on 4 °C condition for 60 min, and protein expression was induced by adding 0.5 mM IPTG, then incubated for an additional 12 h at 23 °C.

Cells were harvested by centrifugation and resuspended in BufferA-HNL (10 mM potassium phosphate (pH 7.0) and 50 mM NaCl). After sonication, the supernatant was collected by centrifugation at 29,300*g* for 40 min. The collected solution was loaded onto a HisTrap HP column (Cytiva) pre-equilibrated with BufferA-HNL. After the column was washed with BufferA-HNL containing 70 mM imidazole, the HNLs were eluted using BufferA-HNL containing 300 mM imidazole. Eluted samples were concentrated to 500 μL with an Amicon Ultra-15 centrifugal filter (Merck Millipore) and further purified by size-exclusion chromatography on a Superdex 200 Increase 10/300 GL column (Cytiva) equilibrated with BufferA-HNL. Purity was confirmed by SDS-PAGE, and the protein concentration was determined by measuring absorbance at 280 nm (NanoDrop One, Thermo Fisher Scientific). Expression levels were estimated from the quantity of purified protein obtained after size-exclusion chromatography (Table S6).

### Analysis of Enzymatic Properties of GanHNLs

Enzymatic activity of the HNLs was measured by quantifying time-dependent benzaldehyde production using a colorimeter method, and all experiments were performed in at least triplicate. The assay buffer consisted of 100 mM sodium citrate (pH 5.5) and 0.1–12 mM racemic mandelonitrile. The reaction was initiated by adding purified HNL to a cuvette containing the assay buffer. Initial velocities were determined by monitoring the time-dependent absorbance change at 280 nm (ε_280_ = 1376 M^–1^·cm^–1^) with an UV-2600i spectrometer (Shimadzu). Kinetic parameters were obtained by fitting the initial velocity data to the Michaelis–Menten equation via a nonlinear least-squares method in ORIGIN software (Figure S2A), and the results are summarized in Table S7.

Thermal stability of the HNLs was assessed by PEAQ-DSC (Malvern Panalytics). Samples of 36.6–398 μM purified protein in BufferA-HNL were heated from 50 to 100 °C at a rate of 90 °C per hour (Figure S2B). The thermodynamic parameters derived from the DSC isotherms are provided in Table S8.

### Design of GanTDHs by GArNet

Two artificial l-threonine 3-dehydrogenases (TDHs) were designed with GArNet following the procedures. The input data were first prepared: structure of TDH from *C. necator* (CnTDH, PDB ID: 3WMX) which was energy-minimized by PyRosetta^27^ and one of sequence library containing total 403 homologue sequences generated in a previous study (the VLYM library^48^). Utilizing the input data, mutations that improve the Rosetta energy unit (REU), and both REU and HiSol values were selected and introduced by GArNet. Here, the detailed calculation conditions, including input parameters (*n*, *m* and *k*), are shown in Table S9. Finally we obtained two TDHs, GanTDH-RE5p and GanTDH-REHi5p (Table S10). Sequence identities among the TDHs are shown in Table S11.

### Overexpression and Purification of GanTDHs

A gene encoding the two GanTDHs (GanTDH-RE5p and GanTDH-REHi5p; Table S10) was synthesized by GenScript with codon optimization for *E. coli* and subcloned into the pET15b vector at the *Nde*I and *Bam*HI restriction sites. Plasmids encoding CnTDH were obtained from a previous study.^41^ All constructs were transformed into BL21­(DE3) cells, which were grown in 1 L of LB medium containing 50 μg/mL carbenicillin at 37 °C until the optical density at 600 nm (OD600) reached 0.6–0.8. Cultures were then placed on 4 °C conditions for 60 min, induced with 0.5 mM IPTG, and incubated for an additional 12 h at 23 °C. Cells were harvested by centrifugation, resuspended in Buffer A-TDH (10 mM potassium phosphate [pH 7.0] and 50 mM NaCl), and then lysed by sonication. The supernatant obtained after centrifugation at 29,300*g* for 40 min was loaded onto a HisTrap HP (Cytiva) column equilibrated with BufferA-TDH. The column was washed with BufferA-TDH containing 70 mM imidazole, and proteins were eluted with BufferA-TDH containing 300 mM imidazole. Eluted fractions were concentrated to 500 μL using an Amicon Ultra-15 (Merck Millipore) centrifugal filter and further purified by size-exclusion chromatography on a Superdex 200 Increase 10/300 GL column (Cytiva) equilibrated with BufferA-TDH. Protein purity was confirmed by SDS-PAGE, and concentrations were determined by measuring absorbance at 280 nm on a NanoDrop One (Thermo Fisher Scientific). Expression levels were estimated by quantifying the amount of target protein recovered after size-exclusion chromatography (Table S12).

### Analysis of Enzymatic Properties of GanTDHs

The kinetic parameters of the TDHs were determined by using an assay buffer containing 0.1 M Gly-KOH (pH 10.0), 2.5 mM NAD^+^, and 0.0–40 mM l-threonine. Initial velocities were measured by monitoring changes in absorbance at 340 nm, corresponding to NADH production, on an UV-2600i spectrometer (Shimadzu). The molar extinction coefficient of NADH at 340 nm is 6300 M^–1^·cm^–1^. Kinetic parameters were obtained by fitting initial velocity data to the Michaelis–Menten equation via a nonlinear least-squares method in ORIGIN software (Figure S3A), and the results are summarized in Table S13.

Thermal stability of CnTDH, GanTDH-RE5p, and GanTDH-REHi5p was evaluated by PEAQ-DSC (MicroCal, Inc.). Prior to the measurement, each TDH was purified by size-exclusion chromatography in Buffer B-TDH (10 mM potassium phosphate [pH 7.0] and 150 mM NaCl). Samples at 6.9–54 μM were then heated from 40 to 100 °C at a rate of 90 °C per hour (Figure S3B). The resulting thermodynamic parameters are listed in Table S14.

## Supplementary Material



## Data Availability

Data and software for GArNet are available at https://github.com/shognakano/GArNet.

## Notes

Details for algorithm of GArNet, schematic view how to generate the scale-free mutation network, enzyme kinetic plots and DSC isotherms of GanHNLs and GanTDHs, estimation of mutational reproducibility for GaHNL-12gen by GAOptimizer, analysis for mutation reproducibility of GArNet by changing generation and cycle numbers, calculation parameters to design GanHNLs by GArNet, protein sequences of the designed HNLs by GArNet, sequence identity between the native and the designed HNLs, protein-soluble expression level of HNLs by the *E. coli* expression system, enzyme kinetic parameters of HNLs, thermodynamic parameters of HNLs estimated by DSC analysis, calculation parameters to design GanTDHs by GArNet, protein sequences of the designed TDHs by GArNet, sequence identity between the native and the designed TDHs, protein-soluble expression level of TDHs by the *E. coli* expression system, enzyme kinetic parameters of TDHs, and thermodynamic parameters of TDHs estimated by DSC analysis (PDF)
