# Supplementary material for: GArNet: A Genetic Algorithm-Based Protein Redesign Approach to Optimize Mutation Combinations Informed by Network Theory
Source: J Chem Inf Model. 2025 Jun 5;65(12):6331–40. doi: 10.1021/acs.jcim.5c00624 (PMC12798769; doi:10.1021/acs.jcim.5c00624)
Supplement: Supplementary file 1 [file ci5c00624_si_001.pdf]

Supporting Information

GArNet: A genetic algorithm-based protein redesign approach to optimize mutation combinations informed by network theory

Hiroki Ozawa, Shoryu Fujita, Taichi Chisuga and Shogo Nakano*

Graduate Division of Nutritional and Environmental Sciences, University of Shizuoka, 52-1 Yada, Suruga-ku, Shizuoka 422-8526, Japan

*Correspondence to Shogo Nakano (snakano@u-shizuoka-ken.ac.jp)

Keywords: Enzyme engineering, genetic algorithm, protein redesign, rational design

Contents

- **Material & Methods** **S3–S5**

- **Supporting Figures** **S6–S8**

Schematic view how to generate scale-free mutation network S6
Enzyme kinetic plots & DSC isotherms of GanHNLs S7
Enzyme kinetic plots & DSC isotherms of GanTDHs S8

- **Table contents** **S9–S10**

Estimation of mutational reproducibility for GaHNL-12gen by GAOptimizer S9
Analysis for mutation reproducibility of GArNet by changing generation and cycle numbers S10
Calculation parameters to design GanHNLs by GArNet S11
Protein sequences of the designed HNLs by GArNet S12
Sequence identity between the native and the designed HNLs S12
Protein soluble expression level of HNLs by E.coli expression system S13
Enzyme kinetic parameters of HNLs S13
Thermodynamic parameters of HNLs estimated by DSC analysis S13
Calculation parameters to design GanTDHs by GArNet S14
Protein sequences of the designed TDHs by GArNet S15
Sequence identity between the native and the designed TDHs S15
Protein soluble expression level of TDHs by E.coli expression system S16
Enzyme kinetic parameters of TDHs S16
Thermodynamic parameters of TDHs estimated by DSC analysis S16

- **References** **S17**

Material & Methods

Details for algorithm of GArNet

The details including classes and functions of GArNet used to generate the mutated proteins, were shown as follows.

1. **Imports and Initialization**
    - Import standard modules (os, sys, re, random, shutil, copy, time).
    - Import pyrosetta, Bio, numpy, networkx, matplotlib.
    - Initialize pyrosetta with init().
2. **Global Setup**
    - Record the start time for performance measurement.
    - Define various input parameters and flags (e.g., PDB file, library directory, chain name, output log file, generation numbers, cycle numbers, mutation counts, selective pressure flags).
3. **Class: STANDARD_TOOLS**
    - **PDB_TO_SEQ(input_pdb, chain_name)**: Extract residues and residue numbers from a given chain in a PDB file, returning the Pose object.
    - **NULL_ELIMINATE(csv_list)**: Remove empty lines from a given list of strings.
4. **Class: MAFFTtoINTMSAlign**
    - **init(library_directory, stp_residue)**:
        - Randomly select a library file, merge it with the STP sequence.
        - Run MAFFT to obtain alignments.
        - Parse alignment to retrieve labels and sequences (CLASSIFICATION).
        - Count amino acid frequencies per residue position based on the template (AMINO_COUNTER).
        - Write the amino acid frequency matrix (INTMSA format) to an output file.
    - **CLASSIFICATION(mafft_data)**: Parse alignment file to obtain all labels and sequences.
    - **AMINO_COUNTER(all_sequences, stp_index)**: Identify non-gapped STP positions, count amino acids at these positions, compute frequency matrix.
5. **Class: CALCULATION_SCORE**
    - **init(pose, ...)**:
        - Perform energy minimization of the pose (ENERGY_MINIMIZE).

        - Compute REU (Rosetta Energy Units) as a fitness metric.
        - If selective pressure is "HISOL", compute a hydrophobicity-based score (HISOL_SCORE).
    - **ENERGY_MINIMIZE(pose, task_pack_mut, scorefxn)**: Set up and run minimization protocol.
    - **HISOL_LIBRARY(intmsa_data, hisol_residue_index)**: Compute library hydrophobicity profiles.
    - **HISOL_SCORE(...)**: Compute a hydrophobicity difference score for the given pose relative to the library.
6. **Class: MUTATION**
    - **init(...)**:
        - The following three functions would be performed to generate mutated structures of which scores (REU, HiSol and both) are improved. The identical calculation was performed in GAOptimizier [1].
    - **SELECT_RANDOM_SITE(...), RANDOM(...), RECOMBINATION(...)**: Methods to determine which residues to mutate. The details were already reported in previous study.
    - **DISULFIDE_CHECK(...), MUTATE_POSE(...)**: By analyzing structure contained in the Pose object, the program firstly check the existence of disulfide bond. If there is the bond, the sites would be eliminated from mutation candidates.
    - **TOURNAMENT(...)**: Tournament selection was performed to generate the next generation's parents. The selected 30 structures would be the parents.
7. **Input Argument Parsing**
    - Parse command-line arguments for PDB, mutation numbers, directories, chain, generation numbers, cycle numbers, selective pressures (REU, HISOL), and other control flags.
8. **Pre-processing**
    - Validate inputs.
    - Extract STP residue and pose from the input PDB.
    - Calculate mutation-related parameters (e.g., mutation rates).
9. **GAOptimizer-like Loop**
    - If skip not requested:
        - Generate the INTMSA files via MAFFTtoINTMSAlign. Here, the INTMSA file contain amino acid frequency matrix (*N* x 21 matrices)

calculated after MAFFT analysis. Here, the *N* represents residue number of the template.

- Load the INTMSA data.
- Run multiple cycles and generations:
  - For each generation, parent structures are mutated to create child samples.
  - Compute scores (REU/HISOL).
  - Record elite structure and wrote scores in output.log.

10. **Determine Final Mutational Combination**
    - If not skipping the network analysis:
      - **EXTRACT_MUTATION_DATA**: Parse final mutation sets adopted during GAOptimizer cycles.
      - **INITIAL_NETWORK**: Build a network (nodes = mutations, edges = co-occurrence) and write out a python script to draw it.
      - Run the network drawing script.
      - **NETWORK_CUT**: Optionally remove unchanged mutations and refine the network.
      - **SELECT_MUTATIONS**: Rank and select a final set of mutations based on specified criteria (ranking, selection strategy).
      - Run the script to finalize the selection of mutations.
11. **Class: FINAL_MUTATE**
    - Introduce the selected mutations into the original pose.
    - Possibly add or remove mutations from the final set (based on sub_mutations_list).
    - Generate multiple PDBs for each candidate final mutation set.
    - Calculate scores and pick the best final variant.
    - Output the best final mutations and their PDB.
12. **Output and Cleanup**
    - Write final results, including best mutations and scores.
    - Compute and record total program execution time.

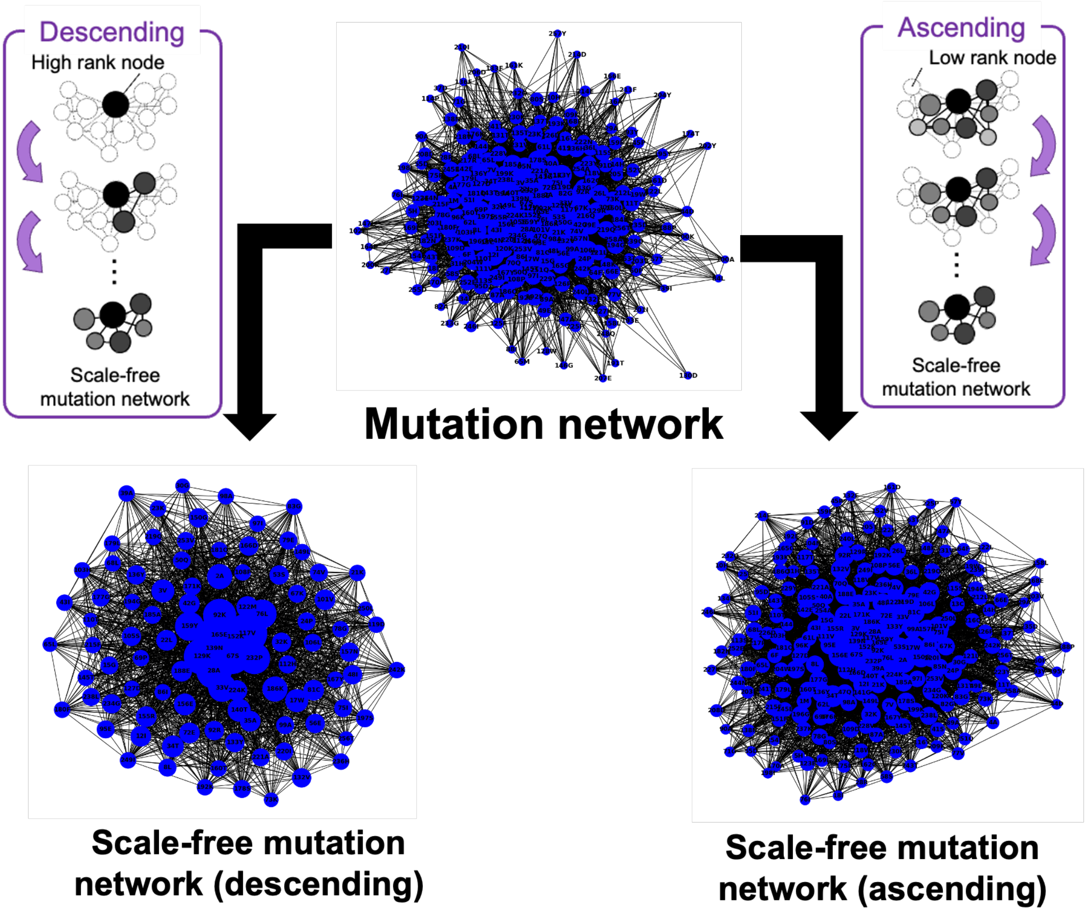

Fig. S1. Schematic view how to generate scale-free mutation network from the mutation network in the Phase II. The mutation network was generated in the design of GanHNL-Hi5p, and the node represents mutation candidates obtained through the phase I calculation. The number of nodes were as follows: 266 (mutation network), 228 (scale-free mutation network (ascending)), and 99 (scale-free mutation network (descending)), respectively.

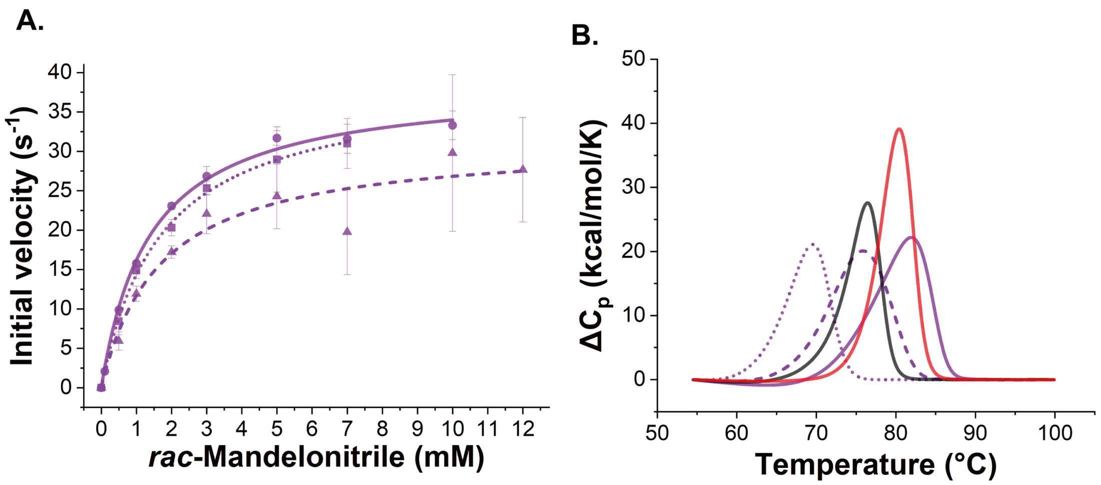

Fig. S2. Enzyme kinetic plots of GanHNLs (A). In the figures, the data for the GanHNL-Hi10, GanHNL-REHi10 and GanHNL-Hi5p were drawn as purple dashed (filled triangle), dotted (filled square), and straight lines (filled circle), respectively. **DSC isotherms of the GanHNLs (B).** The thermodiagrams of MeHNL and GaHNL-12gen were drawn as black and red lines, respectively. All of the measurements were performed at least three replicates, and the data were shown as the mean ± S.D.

*

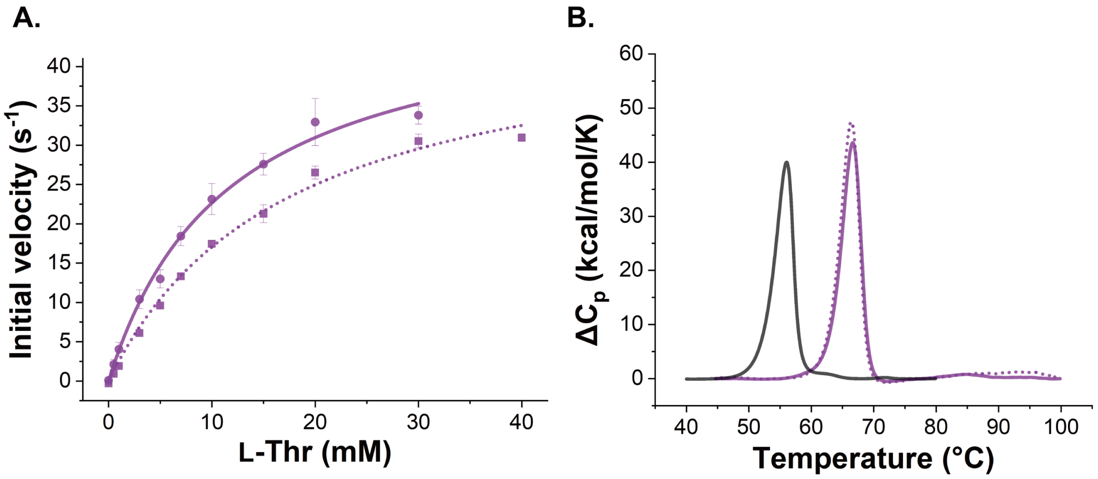

Fig. S3. Enzyme kinetic plots of GanTDHs (A). In the figures, the data for the GanTDH-RE5p and GanTDH-REHi5p were represented as purple straight (filled circle) and dotted lines (filled square), respectively. **DSC isotherms of the GanTDHs (B).** The thermodiagram of CnTDH was drawn as black straight line. All of the measurements were performed by three replicates, and the data were shown as the mean ± S.D.

Table S1. Estimation of mutational reproducibility for designed HNLs by GAOptimizer[a].

Mutant name: GaHNL-12gen (12gen)	
Selection pressure: HiSol score	
1st trial	T3V, N52G, K67S, A82G, **<u>T117V</u>**, R129K, **<u>I139N</u>**, G165E, Q232P (Number of mutations = 9)
2nd trial	V2A, K67S, R92K, **<u>T117V</u>**, E132V, **<u>I139N</u>**, V152K, E166D, P188E (Number of mutations = 9)
3rd trial	R28A, **<u>T117V</u>**, **<u>I139N</u>**, V152K, G165E, P188E (Number of mutations = 6)
Number of overlapping mutations	2
Mutation reproducibility (%)[b]	25.0%

[a]Three variants for each of GaHNL-12gen were designed by GAOptimizer in three independent runs using identical input data to estimate mutational reproducibility. The introduced mutations are listed in tables labeled "1st trial" through "3rd trial."

[b]Mutation reproducibility was calculated by following equation: ((The number of overlapping mutations)*3)/(sum of the mutations for each of the trials)*100.0

Table S2. Analysis for mutation reproducibility of GArNet by changing the number of generations (*n*) and cycles (*m*)[a].

	Mutation reproducibility (%)[b]	
The "*n*" and "*m*" values	GanHNL-Hi10	GanHNL-REHi10
n = 50, m = 10	20.0	30.0
n = 10, m = 50	70.0	50.0
n = 5, m = 100	40.0	50.0
n = 10, m = 100	90.0	80.0

[a]In this analysis, GanHNL-Hi10 and GanHNL-REHi10 were designed by changing generation (*n*) and cycle numbers (*m*).

[b]The Mutational reproducibility was calculated by following equation: ((The number of overlapping mutations)*3)/(sum of the mutations for each of the trials)*100.0.

Table S3. Calculation parameters to design GanHNLs by GArNet.

Sample name		GanHNL-Hi10 (Hi10)	GanHNL-REHi10 (REHi10)	GanHNL-Hi5p (Hi5p)
Template		MeHNL		
Input parameters	Num. of generations (*n*)	10	10	10
	Num. of cycles (*m*)	100	100	100
	Selection pressure	HiSol	HiSol+REU	HiSol
	Num. of mutations (*k*)	10	10	Total 5%[a]
ΔREU		-11.8	-17.0	-8.0
ΔHiSol		-22.3	-18.0	-26.9
Introduced mutations by GArNet[b]		V2A, R28A, K67S, R92K, T117V, R129K, I139N, V152K, G165E, Q232P	R28A, K67S, R92K, T117V, R129K, I139N, G165E, E166D, Q232P	V2A, T3V, R28A, K67S, I76L, R92K, T117V, R129K, I139N, V152K, F159Y, G165E, Q186K, Q232P
Mutation Number		10	9	14
Mutation reproducibility (%)[c]		90.0	80.0	73.5

[a]The number of mutations (*k*) were set to be total 5% of sequence length of MeHNL.

[b]The mutations (total 8 mutations) that would be confirmed in GaHNL-12gen designed by GAOptimizer in previous work were shown as underline.

[c]The mutation reproducibility was calculated by adopting the identical procedure written in Table S1. To calculate the reproducibility, three trials of the enzyme design by GArNet were performed.

Table S4. Protein sequences of the designed HNLs by GArNet

>GanHNL-Hi10 (Hi10)
MATAHFVLIHTICHGAWIWHKLKPALEAAGHKVTALDMAASGIDPRQIEQINSFDEYSEPL
LTFLESLPQGEKVIIVGESCAGLNIAIAADKYVDKIAAGVFHNSLLPDTVHSPSYVVEKLLE
SFPDWKDTEYFTFTNNTGETITTMKLGFKLLRENLFTKCTDEEYELAKMVMRKGSLFQN
VLAQRPKFTEKGYGSIKKVYIWTDQDKIFLPDFQRWQIANYKPDKVYQVPGGDHKLQLT
KTEEVAHILQEVADAYA

>GanHNL-REHi10 (REHi10)
MVTAHFVLIHTICHGAWIWHKLKPALEAAGHKVTALDMAASGIDPRQIEQINSFDEYSEPL
LTFLESLPQGEKVIIVGESCAGLNIAIAADKYVDKIAAGVFHNSLLPDTVHSPSYVVEKLLE
SFPDWKDTEYFTFTNNTGETITTMKLGFVLLRENLFTKCTDEDYELAKMVMRKGSLFQN
VLAQRPKFTEKGYGSIKKVYIWTDQDKIFLPDFQRWQIANYKPDKVYQVPGGDHKLQLT
KTEEVAHILQEVADAYA

>GanHNL-Hi5p (Hi5p)
MAVAHFVLIHTICHGAWIWHKLKPALEAAGHKVTALDMAASGIDPRQIEQINSFDEYSEPL
LTFLESLPQGEKVILVGESCAGLNIAIAADKYVDKIAAGVFHNSLLPDTVHSPSYVVEKLLE
SFPDWKDTEYFTFTNNTGETITTMKLGFKLLRENLYTKCTDEEYELAKMVMRKGSLFQN
VLAKRPKFTEKGYGSIKKVYIWTDQDKIFLPDFQRWQIANYKPDKVYQVPGGDHKLQLT
KTEEVAHILQEVADAYA

Table S5. Sequence identity between the native and the designed HNLs[a]

	MeHNL	12gen	Hi10	REHi10	Hi5p
MeHNL	100%	96.9%	96.1%	96.5%	94.6%
12gen	96.9%	100%	97.7%	97.3%	96.9%
Hi10	96.1%	97.7%	100%	98.8%	98.4%
REHi10	96.5%	97.3%	98.8%	100%	97.3%
Hi5p	94.6%	96.9%	98.4%	97.3%	100%

[a]The sequence identity was cited from the output of ClustalW titled “Percent identity matrix”[2].

Table S6. Protein soluble expression level of HNLs by *E.coli* expression system.

Sample Name	mg/L
Control[a]	
MeHNL	12.8 ± 0.7
GaHNL-12gen	142.3 ± 5.8
In this study	
GanHNL-Hi10	55.9 ± 3.3
GanHNL-REHi10	22.5 ± 5.3
GanHNL-Hi5p	156.5 ± 17.7

[a]The data about MeHNL and GaHNL-12gen were cited from the reference[1].

Table S7. Enzyme kinetic parameters of HNLs.

	k_{cat}	K_m	k_{cat}/K_m
Sample name	s^{-1}	mM	s^{-1} mM^{-1}
Control[a]			
MeHNL	34.5	5.2	6.7
GaHNL-12gen	31.0 ± 0.3	1.7 ± 0.1	18.2 ± 1.1
In this study			
GanHNL-Hi10	31.3 ± 2.6	1.7 ± 0.5	18.4 ± 5.6
GanHNL-REHi10	38.7 ± 0.8	1.7 ± 0.1	22.8 ± 1.4
GanHNL-Hi5p	38.7 ± 0.8	1.4 ± 0.1	27.6 ± 2.1

[a]The parameters were cited from the reference. In the reference, our groups measured the parameters under the identical condition [1].

Table S8. Thermodynamic parameters of HNLs estimated by DSC analysis

	T_m (°C)	ΔH_{cal} (kcal/mol)	ΔH_{VH} (kcal/mol)
Control[a]			
MeHNL	75.4 ± 0.5	131 ± 23	157 ± 14
GaHNL-12gen	80.0 ± 0.0	214 ± 6.0	184 ± 2.6
In this study			
GanHNL-Hi10	74.4 ± 1.0	186 ± 5.6	93.2 ± 9.6
GanHNL-REHi10	67.5 ± 1.4	156 ± 18	124 ± 11
GanHNL-Hi5p	81.1 ± 0.0	152 ± 23	121 ± 1.0

[a]The parameters were cited from the reference. In the reference, our groups measured the parameters under the identical condition [1].

Table S9. Calculation parameters to design GanTDHs by GArNet.

Sample name		GanTDH-RE5p (RE5p)	GanTDH-REHi5p (REHi5p)
Template		CnTDH	
Input parameters	Num. of generations (*n*)	10	10
	Num. of cycles (*m*)	100	150
	Selection pressure	REU	HiSol+REU
	Num. of mutations (*k*)	Total 5%	Total 5%
ΔREU		-47.4	-43.5
ΔHiSol		-27.5	-42.1
Introduced mutations by GArNet[c]		V8I, V43E, R64K, G66K, A84N, A126K, G127E, N158K, D188E, T195K, K204S, E207T, P215D, E234V, G260D, Q262E, D279Q, A294P, G297D	N11C, R28N, V43E, A59E, A84N, A126K, G127E, A157N, N158K, H174Y, D188E, T195K, K204S, E207T, P215D, E234V, A288K, A294P, G297D, A307K
Mutation Number		19	20
Mutation reproducibility (%)[b]		60.0	60.0

[a]The number of mutations (*k*) were set to be total 5% of sequence length of CnTDH.

[b]The mutation reproducibility was calculated by adopting the identical equation written in Table S1. To calculate the reproducibility, three trials of the enzyme design by GArNet were performed.

[c]The mutations that were commonly confirmed in both RE5p and REHi5p were highlighted as underline.

Table S10. Protein sequences of the designed TDHs by GArNet

>GanTDH-RE5p (RE5p)
GKPKILIIGANGQIGSELALALAERYGRTNVITSDVVPTGRHEHLTHEMLNATDRGELATV
VEKHKITQVYLLAAALSATGEKNPQWAWNLNMTSLLNVLELARQTGLERVFWPSSIAAF
GPTTPKEQTPQKTVMEPTTVYGISKQAGEGWCRWYHAKHGVDVRSVRYPGLISHKTP
PGGGTTDYAVEIFHAAVKGEPYTCFLSEDTALPMMYMDDAIRATIELMEAPADKLSVRGS
YNIAGMSFTPAQIAAAIREQVPDFEIRYEPDYRQAIAQGWPQSIDDSVARADWGWKPQY
DLKEMVADMLANLK

>GanTDH-REHi5p (REHi5p)
GKPKILIVGACGQIGSELALALAERYGNTNVITSDVVPTGRHEHLTHEMLNATDRGELET
VVERHGITQVYLLAAALSATGEKNPQWAWNLNMTSLLNVLELARQTGLERVFWPSSIAA
FGPTTPKEQTPQKTVMEPTTVYGISKQAGEGWCRWYHNKHGVDVRSVRYPGLISYKT
PPGGGTTDYAVEIFHAAVKGEPYTCFLSEDTALPMMYMDDAIRATIELMEAPADKLSVRG
SYNIAGMSFTPAQIAAAIREQVPGFQIRYEPDYRQAIAQGWPDSIDDSVARKDWGWKPQ
YDLKEMVADMLKNLK

Table S11. Sequence identity between the native and the designed TDHs[a]

	CnTDH	RE5p	REHi5p
CnTDH	100%	93.9%	93.5%
RE5p	93.9%	100%	95.8%
REHi5p	93.5%	95.8%	100%

[a]The sequence identity was cited from the output of Clustalw titled “Percent identity matrix”[2].

Table S12. Protein soluble expression level of TDHs by *E.coli* expression system.

Sample Name	mg/L
Control[a]	
CnTDH	64.1 ± 15.5
In this study	
GanTDH-RE5p	81.2 ± 19.2
GanTDH-REHi5p	36.8 ± 2.7

[a]The data about TDH was cited from the reference [1].

Table S13. Enzyme kinetic parameters of TDHs.

	k_{cat}	K_m	k_{cat}/K_m
Substrate	s^{-1}	mM	s^{-1} mM^{-1}
Control[a]			
CnTDH	118.9 ± 6.0	17.4 ± 1.6	6.8 ± 0.7
In this study			
GanTDH-RE5p	48.9 ± 2.6	11.6 ± 1.4	4.2 ± 0.6
GanTDH-REHi5p	46.6 ± 2.4	17.4 ± 1.9	2.7 ± 0.3

[a]The parameters were cited from the reference [1]. In the reference, our groups measured the parameters under the identical condition [1].

Table S14. Thermodynamic parameters of TDHs estimated by DSC analysis

	T_m (°C)	ΔH_{cal} (kcal/mol)	ΔH_{VH} (kcal/mol)
Control[a]			
CnTDH	55.7 ± 0.0	180 ± 0.9	237 ± 1.4
In this study			
GanTDH-RE5p	66.4 ± 0.0	170 ± 5.0	230 ± 3.0
GanTDH-REHi5p	66.1 ± 0.1	190 ± 5.0	228 ± 3.8

[a]The parameters were cited from the reference [1]. In the reference, our groups measured the parameters under the identical condition [1].

References

[1] Ozawa, H., Unno, I., Sekine, R., Chisuga, T., Ito, S., and Nakano, S. (2024) Development of evolutionary algorithm-based protein redesign method, *Cell Reports Physical Science 5*, 101758.

[2] Thompson, J. D., Higgins, D. G., and Gibson, T. J. (1994) CLUSTAL W: improving the sensitivity of progressive multiple sequence alignment through sequence weighting, position-specific gap penalties and weight matrix choice, *Nucleic Acids Res 22*, 4673-4680.
